# Screening, Identification, and Degradation Mechanism of Polyester Fiber-Degrading Bacteria

**DOI:** 10.3390/microorganisms14010207

**Published:** 2026-01-16

**Authors:** Zixuan Chen, Jing Tang, Shengjuan Peng, Qin Chen, Jianfeng Bai, Weihua Gu

**Affiliations:** School of Resources and Environmental Engineering, Shanghai Polytechnic University, Shanghai 201209, China

**Keywords:** polyethylene terephthalate, polyester fiber, microbial degradation, *Bacillus*, degradation mechanism

## Abstract

Polyester fibers are extensively used in textiles, packaging, and industrial applications due to their durability and excellent mechanical properties. However, high-crystallinity polyester fibers represent a major challenge in plastic waste management due to their resistance to biodegradation. This study evaluated the biodegradation potential of environmental *Bacillus* isolates, obtained from mold-contaminated black bean plastic bags, toward polyethylene terephthalate (PET) and industrial-grade polyester fibers under mesophilic conditions. Among thirteen isolates, five (*Bacillus altitudinis* N5, *Bacillus subtilis* N6, and others) exhibited measurable degradation within 30 days, with mass losses up to 5–6% and corresponding rate constants of 0.04–0.05 day^−1^. A combination of complementary characterization techniques, including mass loss analysis, scanning electron microscopy (SEM), gel permeation chromatography (GPC), and gas chromatography/mass spectrometry (GC/MS), together with Fourier-transform infrared spectroscopy (FTIR), thermogravimetric/differential scanning calorimetry (TGA/DSC), and water contact angle (WCA) analysis, was employed to evaluate the biodegradation behavior of polyester fibers. Cross-analysis of mass loss, surface morphology, molecular weight reduction, and degradation products suggests a surface erosion-dominated degradation process, accompanied by ester-bond hydrolysis and preferential degradation of amorphous regions. FTIR, TGA/DSC, and WCA analyses further reflected chemical, thermal, and surface property changes induced by biodegradation rather than directly defining the degradation mechanism. The findings highlight the capacity of mesophilic *Bacillus* species to partially depolymerize polyester fibers under mild environmental conditions, providing strain resources and mechanistic insight for developing low-energy bioprocesses for polyester fiber waste management.

## 1. Introduction

Polyethylene terephthalate (PET) is one of the most widely used synthetic fibers due to its excellent mechanical properties, chemical resistance, and ease of processing. It is commonly utilized in the apparel, home furnishings, and industrial sectors, making it a significant alternative to natural fibers. The market value of polyester fibers is projected to reach USD 111.61 billion by 2025 and USD 191.4 billion by 2032 [1]. However, the inherent chemical stability of PET also presents substantial environmental challenges. PET fibers are highly resistant to degradation in natural environments, leading to the accumulation of large quantities of discarded textiles and plastic waste in terrestrial and marine ecosystems. This has resulted in widespread microplastic pollution, which poses serious risks to both ecosystems and human health [2]. Thus, the development of effective strategies for managing and treating polyester fiber waste has become an urgent priority.

Current methods for processing polyester fibers include physical recycling, chemical recycling, energy recovery, biodegradation, landfilling, and upcycling through innovative applications. Mechanical recycling, a simple method of circular utilization, recovers material through mechanical decomposition and crushing processes, and has established a relatively well-developed global infrastructure [3]. However, the external forces applied during mechanical crushing and the synergistic effect of detergents used in the washing process can accelerate the aging and degradation of fibers. This results in a significant decrease in their polymerization degree, weakening their mechanical properties, and affecting the quality of recycled yarn [4]. Compared to virgin PET, mechanically recycled PET may discolor, become cloudy, and release more plastic particles, making it unsuitable for many packaging applications [5]. Chemical recycling, through depolymerization and repolymerization, can produce materials with performance equivalent to virgin PET, thus serving as an important method for polyester fiber recycling. However, the use of strong acids or bases as solvents in the depolymerization process can cause severe equipment corrosion and generate large amounts of waste during the post-processing stage [4]. Incineration has the highest global warming potential (GWP) and primary energy consumption. Assessments of related technologies, particularly combined heat and power (CHP) incineration plants, have shown significant environmental concerns [6]. In 2015, the plastics industry accounted for 4.5% of global greenhouse gas emissions [7]. Landfilling remains one of the primary methods for disposing of waste textiles worldwide, with approximately 87% of waste textiles ultimately ending up in landfills [8]. However, due to their high crystallinity and hydrophobic nature, polyester fibers are highly resistant to both biological and abiotic degradation under landfill or natural environmental conditions [9]. Additionally, landfilling consumes significant land area, and improper management may lead to soil contamination and serve as a significant source of microplastics in the environment [10].

From the perspective of biological treatment, the intrinsic physicochemical properties of PET further limit its degradability. High crystallinity, strong hydrophobicity, and low surface accessibility hinder microbial attachment and restrict the interaction between depolymerizing enzymes and the polymer chains. Therefore, pretreatment of PET waste is often regarded as an important prerequisite for effective biodegradation. Common pretreatment strategies, including mechanical milling, alkaline or acidic treatment, thermal processing, and surface oxidation, are applied to reduce crystallinity, increase surface roughness, or introduce polar functional groups, thereby improving enzymatic accessibility and degradation efficiency. However, many of these pretreatment processes are energy-intensive, chemically demanding, or environmentally burdensome, which may restrict their large-scale application and overall sustainability. In this context, increasing attention has been directed toward identifying microorganisms capable of initiating PET degradation under mild conditions with limited or no pretreatment, particularly for high-crystallinity polyester fibers commonly present in textile waste.

In recent years, biodegradation has emerged as a promising solution for polyester fiber waste treatment due to its environmentally friendly and mild reaction conditions. Previous studies have demonstrated that a limited number of microorganisms, including *Ideonella sakaiensis*, *Comamonas testosteroni*, *Pseudomonas putida*, and several fungal species, are capable of degrading PET through the secretion of hydrolytic enzymes such as PETase, MHETase, chitinase, and lipase, which catalyze ester bond cleavage and generate low-molecular-weight products including terephthalic acid (TPA) and ethylene glycol (EG) [11,12]. Beyond individual strains, PET degradation has also been reported in complex microbial communities, where genera such as *Thalassospira*, *Nitratireductor*, *Nocardioides*, *Muricauda*, *Owenweeksia*, *Alcanivorax* and *Pseudomonas* were identified as dominant contributors, achieving PET removal efficiencies of 1.8–16.2% over two months [13]. In addition, several high-performance systems have been described, including *Gordonia* sp. *CN2K*, which degraded over 40% of PET within 45 days [14], and thermostable PET hydrolases such as CaPETase, which exhibited substantial activity at ambient temperature [15]. PET biodegradation has also been observed in textile-related materials, such as mooring ropes degraded by Lipomyces yeasts, accompanied by changes in polymer crystallinity [16]. *Bacillus* species have attracted increasing attention due to their metabolic versatility and capacity to secrete diverse extracellular enzymes involved in plastic degradation. Various *Bacillus* strains have been reported to degrade different synthetic polymers, including LDPE, PLA, PS, PP, PVC, and PBAT [17,18,19,20,21,22], as well as blended plastics and additives such as starch-based plastics and bisphenol A (BPA) [23,24]. These findings highlight the broad plastic-degrading potential of this genus.

Despite these advances, most PET-degrading microorganisms and enzymes reported to date originate from thermophilic environments, with optimal degradation temperatures typically between 50 °C and 70 °C. Nevertheless, mesophilic PET biodegradation has also been demonstrated. For example, *Ideonella sakaiensis* was shown to almost completely degrade low-crystallinity PET under mild conditions (30 °C, pH 7.0) within 42 days [25], supporting the feasibility of enzyme- and microbe-mediated PET depolymerization under environmentally relevant conditions. Such enzyme-catalyzed processes have therefore emerged as promising strategies for sustainable plastic recycling and upcycling [26].

Although significant progress has been made in microbial and enzymatic PET degradation, most existing studies have primarily focused on low-crystallinity PET films or powders, thermophilic conditions, or genetically engineered enzymes operating under highly optimized laboratory settings. Consequently, the biodegradation behavior of high-crystallinity, industrial-grade polyester fibers under mesophilic and environmentally relevant conditions remains insufficiently understood. In this study, we report the isolation and characterization of two *Bacillus* strains capable of initiating the biodegradation of polyester fibers under mild conditions with minimal pretreatment. By integrating degradation performance evaluation with surface morphology analysis, molecular weight characterization, and degradation product identification, this work provides a systematic assessment of polyester fiber biodegradation and supports a surface erosion-dominated degradation pathway.

Overall, this study contributes new insight by linking microbial screening, degradation behavior, and mechanistic interpretation for high-crystallinity polyester fibers, thereby providing both microbial resources and mechanistic understanding relevant to the development of low-energy and sustainable strategies for polyester fiber waste management.

## 2. Materials and Methods

### 2.1. Strain Origin

Environmental samples consisting of plastic waste contaminated with moldy black beans were collected and used for microbial isolation. The target strains were domesticated using polyethylene terephthalate (PET) particles as the sole carbon source in mineral salt medium (MSM). The cultures were incubated at 30 °C with continuous agitation (140 rpm) for 40 days. Thirteen morphologically distinct strains exhibiting stable growth were obtained and preserved for further analyses.

The isolates cultured in PDA liquid medium were designated C1–C6, and those in BPM medium as N1–N7. Genomic DNA was extracted using a commercial bacterial genomic DNA kit (Wuhan Namag Bio, Wuhan, China). The 16S rRNA gene was amplified with primers 27F (5′-AGAGTTTGATCCTGGCTCAG-3′) and 1492R (5′-TACGGCTACCTTGTTACGACTT-3′). PCR products were purified and sequenced (BGI, Shenzhen, China), and the resulting sequences were compared with reference sequences in the NCBI database using the BLASTn program available on the NCBI website (https://blast.ncbi.nlm.nih.gov/Blast.cgi), accessed on 5 June 2024, for taxonomic identification.

### 2.2. Experimental Materials

Materials: PET pellets (Adamas, Shanghai, China), polyester fiber fabric (cut into 1 × 1 cm pieces).

Media: BPM (peptone 10.0 g/L, sodium chloride 5.0 g/L, beef extract 3.0 g/L, pH 7.2 ± 0.1);

PDA (potato dextrin 12.0 g/L, glucose 20.0 g/L, agar 14.0 g/L, pH 5.6 ± 0.2);

MSM (potassium dihydrogen phosphate 3.0 g/L, magnesium sulfate heptahydrate 0.1 g/L, etc., pH 7.5 ± 0.1).

Prior to incubation, all culture media and experimental vessels were sterilized by autoclaving.

### 2.3. Stepwise Screening of Polyester-Degrading Bacterial Strains

#### 2.3.1. Initial Screening Using PET Particles

The PET-degrading ability of the isolated strains was preliminarily evaluated using PET particles as the sole carbon source. Briefly, thirteen candidate strains were separately inoculated into MSM liquid medium containing 1% (*w_t_*/*vol*) PET particles at an inoculation ratio of 6% (*v*/*v*). An uninoculated MSM medium containing PET particles served as the control (CK). All cultures were incubated at 30 °C and 140 rpm for 30 days, with three independent replicates for each treatment.

After incubation, PET particles were collected, washed, dried to constant weight, and the weight loss percentage was determined gravimetrically. The PET particle weight loss was used as the primary criterion for the preliminary screening of PET-degrading strains.

#### 2.3.2. Re-Screening Using Polyester Fiber Fabric

Based on the preliminary screening results, nine strains (C2, C6, and N1–N7) exhibiting measurable PET particle degradation were selected for secondary screening using commercial polyester fiber fabric as the substrate. Polyester fabric was cut into 1 × 1 cm pieces, sterilized with 75% ethanol followed by UV irradiation, and added to MSM medium at a concentration of 1% (*w_t_*/*vol*). Uninoculated medium containing polyester fiber served as the control (CK).

Each strain was inoculated at 6% (*v*/*v*), and the cultures were incubated at 30 °C and 140 rpm for 30 days, with three parallel replicates per group. After incubation, the polyester fibers were recovered, washed, dried to constant weight, and the weight loss percentage was determined. The biodegradation performance of each strain was evaluated based on polyester fiber weight loss.

### 2.4. Study on the Biodegradability of Polyester Fibers

#### 2.4.1. Construction of Degradation Systems

Five strains were separately activated in BPM medium at 30 °C and 140 rpm for 3 days until an OD_600_ of 1.0 was reached. The strains were then inoculated into MSM medium containing 1% (*w_t_*/*vol*) polyester fiber at an inoculum concentration of 6% (*v*/*v*), with an uninoculated group serving as the control (CK). Cultures were incubated at 30 °C and 140 rpm for 30 days, with three replicates per group.

#### 2.4.2. Material Characterization

The biodegradation performance of polyester fibers was evaluated through microbial growth, weight loss, molecular weight variation, mechanical properties, surface morphology, hydrophilicity, and thermal behavior, as described below.

(1) Analysis of Microbial Growth

The OD value at 600 nm was measured at regular intervals over the 30-day incubation period using a full-wavelength microplate reader (Multiskan 1550, Thermo Fisher Scientific, Waltham, MA, USA) to construct microbial growth curves.

(2) Weight Loss

After treatment with 2% SDS and freeze-drying, the fibers were weighed, and the degree of degradation was determined by comparing the weight of the dry PET before and after degradation [27]. The weight loss percentage due to degradation was calculated using the following Equation (1):

(1) $$Weight\;loss\;rate=\frac{W_{0}\;-W}{W_{0}}\;\times \;100\%$$

W_0_—initial weight (g)

W—remaining weight (g)

Using a first-order kinetic model, the removal rate constant (K) for PET particles was determined according to Equation (2):

(2) $$K=\;-\;\frac{1}{t}(\ln\frac{W}{W_{0}})$$

K—polymer removal rate constant (d^−1^);

t—degradation time (d).

The half-life (t_1/2_) was calculated from the removal rate constant using Equation (3):

(3) $$t_{\frac{1}{2}}\;=\;\frac{\ln2}{K}$$

(3) GPC analysis

Gel permeation chromatography (GPC) analysis was performed to determine the molecular weight changes of polyester fibers before and after biodegradation (Agilent 1260, Agilent Technologies, Santa Clara, CA, USA). Polyester samples were dissolved in hexafluoroisopropanol (HFIP) and filtered to remove insoluble residues prior to analysis. Polymethyl methacrylate (PMMA) standards were used for calibration. The number-average molecular weight (M_n_) and weight-average molecular weight (M_w_) were calculated based on the calibration curve.

(4) Mechanical properties

Tensile strength was measured using an electronic universal testing machine (ASG-X1KN, Shimadzu, Kyoto, Japan), with a specimen grip spacing of 5 mm and a tensile speed of 100 mm/min.

(5) SEM analysis

The surface morphology of the fibers was observed using a field-emission scanning electron microscope (S4800, Hitachi, Tokyo, Japan). The samples were gold-coated and analyzed at an acceleration voltage of 15 kV.

(6) Water contact angle

The water contact angle was measured using a dynamic contact angle meter (JY-82C, Chengde Dingsheng Testing Machine Co., Ltd., Chengde, China) with the sessile drop method. A 4 μL droplet of deionized water was applied, and five measurements were taken per group.

(7) Thermal properties

Thermal properties were analyzed using a simultaneous thermal analyzer (STA6000, PerkinElmer, Waltham, MA, USA) to generate TG-DTG curves under a nitrogen atmosphere with a heating rate of 10 °C/min up to 800 °C. Differential scanning calorimetry (DSC) analysis was performed using a DSC3 system (Mettler-Toledo, Greifensee, Switzerland). DSC curves were recorded from 130 °C to 300 °C and then cooled back to 130 °C at a heating rate of 10 °C/min. The melting enthalpy (ΔH_m_) of polyester fibers was determined by integrating the area under the melting endothermic peak of the DSC heating curve. The degree of crystallinity (X_c_) was subsequently calculated according to Equation (4), using a reference melting enthalpy for 100% crystalline polyester (${\Delta H}_{m}^{{}^{\circ}}$ = 140 J/g) [28].

(4) $$Xc=\frac{∆H_{m}}{\omega_{\mathrm{PET}}∆H_{m}^{{}^{\circ}}}\times 100\%$$

X_c_—crystallinity, %;

ΔH_m_—melting enthalpy of the test sample, J/g;

$\omega_{\mathrm{PET}}$—mass fraction of polyester fiber, 93%;

$\Delta H_{m}^{{}^{\circ}}$—standard melting enthalpy of fully crystallized polyester fiber, 140 J/g.

(8) FTIR analysis

Fourier transform infrared (FTIR) spectroscopy was used to analyze changes in surface functional groups of polyester fibers before and after microbial degradation. FTIR spectra were collected using an FTIR spectrometer (Lyza 7000, Anton Paar, Graz, Austria) in attenuated total reflectance (ATR) mode. All samples were scanned over the wavenumber range of 4000–500 cm^−1^ with a resolution of 4 cm^−1^, and 32 scans were accumulated for each spectrum. The obtained spectra were baseline-corrected and normalized prior to comparison to identify variations in characteristic absorption bands associated with polyester functional groups.

(9) GC-MS analysis

Gas chromatography–mass spectrometry (GC–MS) was employed to identify degradation products and to investigate molecular-level changes in polyester fibers induced by microbial treatment. Approximately 0.5–1.0 mg of dried fiber sample was subjected to solvent extraction to obtain low-molecular-weight degradation products, followed by GC–MS analysis using an Agilent GC–MS system (Agilent Technologies, Inc., Santa Clara, CA, USA). The extracted samples were injected into the GC system, and the degradation products were separated on a capillary column and analyzed by mass spectrometry in electron ionization (EI) mode at 70 eV. Compound identification was performed by comparing the obtained mass spectra with those in the NIST mass spectral library.

## 3. Results and Discussion

### 3.1. Identification and Stepwise Screening of Polyester-Degrading Bacterial Strains

Thirteen strains capable of stable growth on PET particles were successfully isolated and designated C1–C6 and N1–N7 according to their enrichment media. 16S rRNA gene sequencing followed by BLAST analysis (Table 1) revealed that all isolates belonged to the genus *Bacillus*. Specifically, isolates N5 and N6 showed high sequence similarity (≥99%) to *Bacillus altitudinis* and *Bacillus subtilis*, respectively, whereas C3 and C5 were closely related to *Lysinibacillus* sp. The predominance of *Bacillus* species in the samples may be related to the storage environment of the mold-contaminated black beans, which provides nutrient sources and conditions favoring spore-forming bacteria. These isolates were subsequently subjected to a stepwise screening procedure to evaluate their polyester-degrading potential.

**Figure 1:**
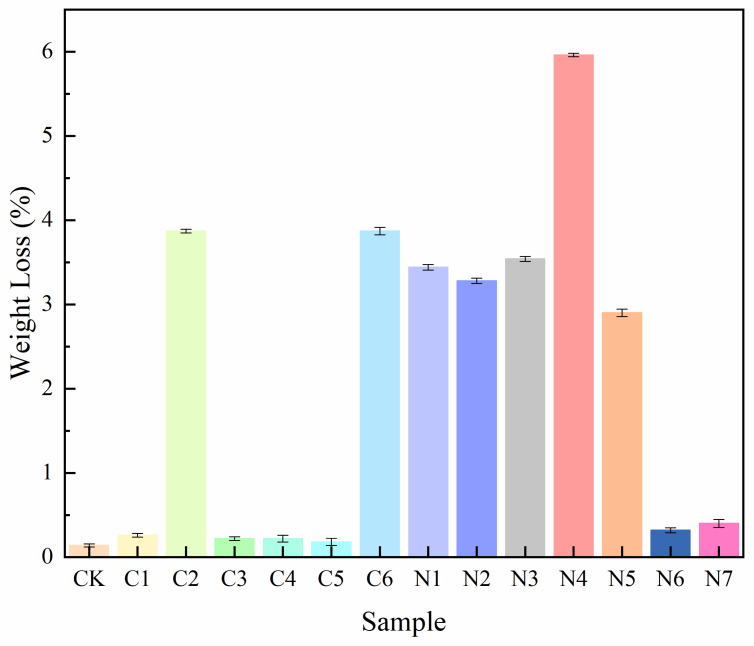
Weight loss of PET particles after 30 days of incubation with different bacterial strains.

In the preliminary screening stage, the PET-degrading capability of all 13 isolates was evaluated using PET particles as the sole carbon source. After 30 days of incubation, measurable PET weight loss was observed for all inoculated treatments, whereas the uninoculated control (CK) exhibited only negligible mass loss (0.14%), confirming that abiotic degradation under the experimental conditions was minimal (Figure 1). Significant differences in PET degradation efficiency were detected among the strains (*p* < 0.05). Specifically, strains C2, C6, N1, N2, N3, N4, and N5 induced relatively higher PET weight loss, with values ranging from 2.90% to 5.96%. Among them, strain N4 exhibited the highest degradation efficiency, achieving a PET weight loss of 5.96%, indicative of strong PET-degrading potential under mesophilic conditions. In contrast, strains C1, C3, C4, C5, N6, and N7 showed limited degradation activity, with weight loss rates below 0.5%.

Kinetic analysis further highlighted strain-dependent differences in PET degradation behavior. Under the action of strain N4, the apparent first-order degradation rate constant reached 0.00205 d^−1^, corresponding to a calculated half-life of 338.12 days for 0.25 g PET particles. In contrast, strain C5 exhibited a markedly lower degradation rate constant (0.00006 d^−1^), with an extended half-life of 11,552.45 days, reflecting extremely limited PET degradation capability. These results further support the superior PET-degrading performance of strain N4 during the preliminary screening stage.

Based on the preliminary screening results, nine strains (C2, C6, N1–N7) exhibiting measurable growth or degradation activity toward PET particles were subjected to a secondary screening using commercial polyester fiber fabric as the substrate, which more closely represents real textile-derived polyester waste. After 30 days of incubation, clear strain-dependent differences in polyester fiber degradation were observed. Strains N3, N4, N5, N6, and N7 demonstrated effective biodegradation activity, with fiber weight loss rates of 2.43%, 3.85%, 2.19%, 2.52%, and 2.14%, respectively. Among them, strain N4 again showed the highest degradation efficiency, with a weight loss approximately 29.6-fold higher than that of the control group (CK), indicating strong adaptability to polyester fiber substrates.

In contrast, strains C2, C6, N1, and N2 exhibited relatively limited degradation performance toward polyester fibers, despite showing moderate PET particle degradation in the preliminary screening. Notably, strain N2 displayed the lowest fiber degradation efficiency, with a weight loss only 6.6 times higher than that of the control. These results suggest that PET particle degradation capacity does not necessarily translate into effective degradation of polyester fibers, likely due to differences in substrate morphology, crystallinity, and additive composition.

Accordingly, strains N3, N4, N5, N6, and N7 were selected as representative and effective polyester fiber-degrading bacteria for subsequent investigations into fiber biodegradation behavior, structural changes, and degradation mechanisms.

### 3.2. Influence of Bacterial Growth on Polyester Fiber Degradation Performance

The growth behavior of the selected bacterial strains was evaluated to clarify its role in polyester fiber biodegradation. As shown in Figure S1, all five strains (N3, N4, N5, N6, and N7) exhibited typical bacterial growth phases, including lag, exponential, and stationary phases, when cultivated in MSM medium containing polyester fibers as the sole carbon source. This confirms that the selected strains were able to adapt to the polyester-based environment and sustain metabolic activity throughout the incubation period. Although all strains demonstrated comparable growth patterns, noticeable differences in biomass accumulation were observed. Strains N5 and N6 entered the exponential growth phase earlier and achieved higher final cell densities than the other strains, whereas N3 and N7 showed relatively lower biomass levels. These differences suggest strain-dependent adaptability and metabolic efficiency toward polyester fiber substrates.

To further examine the relationship between bacterial growth and degradation performance, correlation analysis was conducted between biomass accumulation and polyester fiber weight loss. A significant positive correlation was observed (R^2^ = 0.87, *p* < 0.01), indicating that higher bacterial biomass generally favors more efficient degradation. This trend implies that sufficient cell density is a prerequisite for effective enzymatic attack on the polymer surface.

It should be noted, however, that biomass accumulation alone does not fully determine degradation efficiency. For example, although strain C6 exhibited relatively high biomass during screening experiments, its degradation performance was not among the highest. This observation suggests that factors such as extracellular enzyme activity, enzyme–substrate affinity, and metabolic pathway regulation also play critical roles in polyester fiber biodegradation. Therefore, bacterial growth is considered a supportive but not exclusive factor influencing degradation efficiency.

### 3.3. Study on the Biodegradability of Polyester Fibers

#### 3.3.1. Weight Loss and Degradation Kinetics of Polyester Fibers

**Figure 2:**
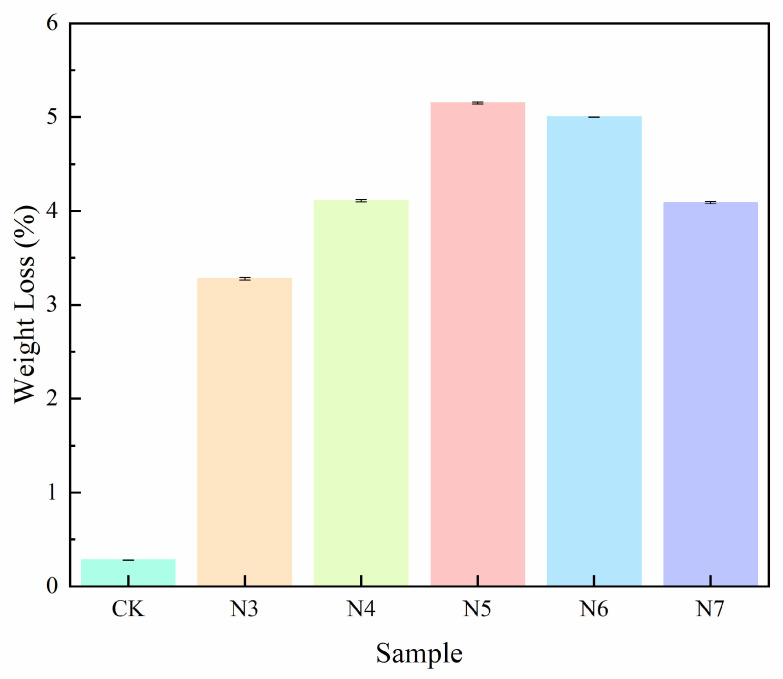
Weight loss of polyester fiber fabrics after 30 days of incubation with different bacterial strains.

To further evaluate the biodegradation performance of the selected strains beyond the screening stage, changes in polyester fiber mass and degradation kinetics were systematically analyzed. As shown in Figure 2 and Table 2, the uninoculated control (CK) exhibited only a marginal weight loss of 0.14%, which can be primarily attributed to non-biological losses during washing and sample handling, confirming the negligible contribution of abiotic degradation under the experimental conditions. After 30 days of incubation with equal inoculum concentrations, distinct strain-dependent differences in polyester fiber degradation were observed. Strains N5 and N6 induced the most pronounced mass loss, with weight loss rates of 5.15% and 5.00%, respectively, indicating a stronger capacity to disrupt the fiber structure. In contrast, strains N4, N7, and N3 caused comparatively lower mass losses (4.11%, 4.09%, and 3.38%, respectively), with N3 showing the weakest degradation performance among the tested strains.

To quantitatively characterize the degradation dynamics, a first-order kinetic model was applied to the weight loss data. Strain N5 exhibited the highest degradation rate constant (k = 0.00176 d^−1^), corresponding to a half-life (t_1/2_) of 393.83 days for 0.125 g polyester fiber, indicating a relatively rapid and sustained degradation process. By comparison, strain N3 displayed a lower degradation rate constant (k = 0.00115 d^−1^) and a markedly longer half-life (602.74 days), reflecting slower degradation kinetics despite observable mass loss.

Overall, the combined analysis of weight loss and kinetic parameters demonstrates that strains N5 and N6 not only promote greater overall mass reduction of polyester fibers but also exhibit more efficient degradation dynamics, suggesting their superior potential for polyester fiber biodegradation under the tested conditions.

#### 3.3.2. Changes in Molecular Weight and Mechanical Properties

**Figure 3:**
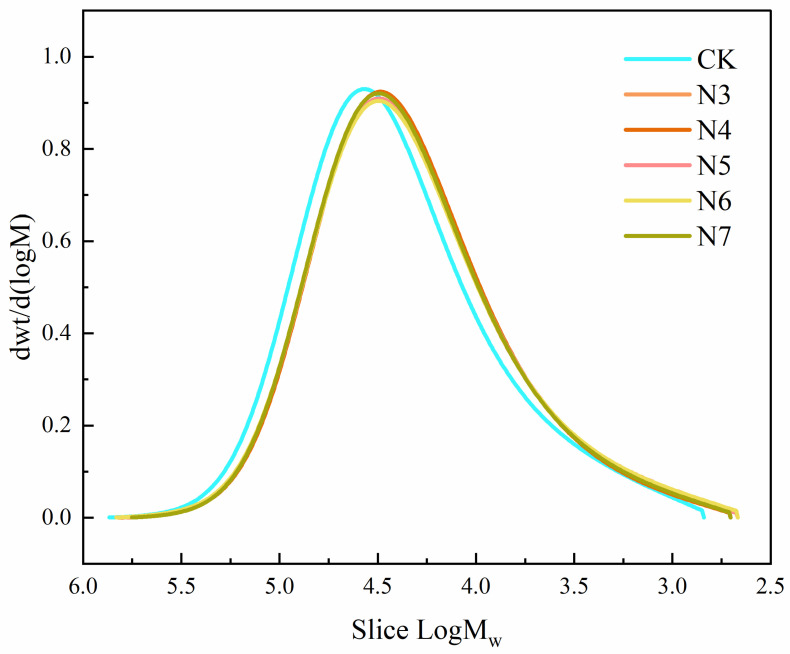
Molecular weight distribution curves of polyester fiber samples after bacterial degradation.

Molecular weight distribution analysis of the polyester fiber samples after bacterial degradation (Figure 3) revealed that the GPC curves of the samples treated with specific bacterial strains exhibited a visually apparent and consistent shift toward the lower molecular weight region, compared to the control (CK). Based on this shift, it can be inferred that polyester fibers underwent partial depolymerization during the biodegradation process.

**Figure 4:**
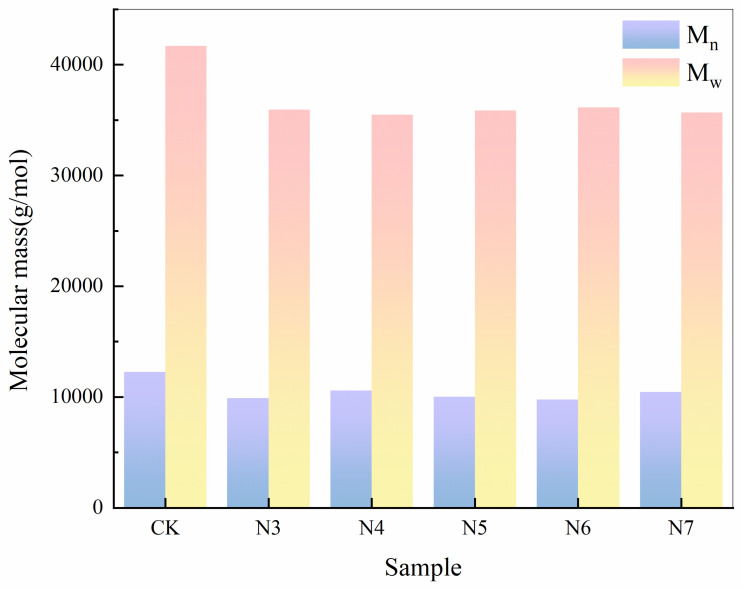
Changes in molecular weight (M_n_ and M_w_) of polyester fibers degraded by different *Bacillus* strains.

To further support this observation, the average molecular weights of the degraded polyester fabrics were quantitatively determined, as shown in Figure 4. Gel permeation chromatography (GPC) analysis showed that, after treatment with all five strains of bacteria, the number-average molecular weight (M_n_) and weight-average molecular weight (M_w_) of the polyester fibers exhibited measurable decreases compared with the control. Among these, strain N6 exhibited a relatively greater reduction in M_n_ (from 28,600 g/mol to 22,800 g/mol, a 20.3% decrease) and M_w_ (from 45,200 g/mol to 38,300 g/mol, a 15.4% decrease), followed by strain N5 (18.2% and 13.7% decreases, respectively). These reductions were consistently higher than those observed for the other strains (*p* < 0.05). The increase in the molecular weight distribution index (PDI) from 1.58 to 1.72–1.89 indicates non-uniform chain scission of fiber molecules and a rise in the proportion of low–molecular–weight fragments, consistent with the random nature of enzymatic hydrolysis during biodegradation [29].

Taken together, these results indicate that, under the action of the selected bacterial strains, the long-chain molecular structure of polyester fibers underwent progressive chain scission. This process led to a measurable reduction in molecular weight and, based on the observed changes in molecular weight distribution, suggests that polyester degradation proceeded as a dynamic transformation from higher-molecular-weight fractions toward lower-molecular-weight species.

**Figure 5:**
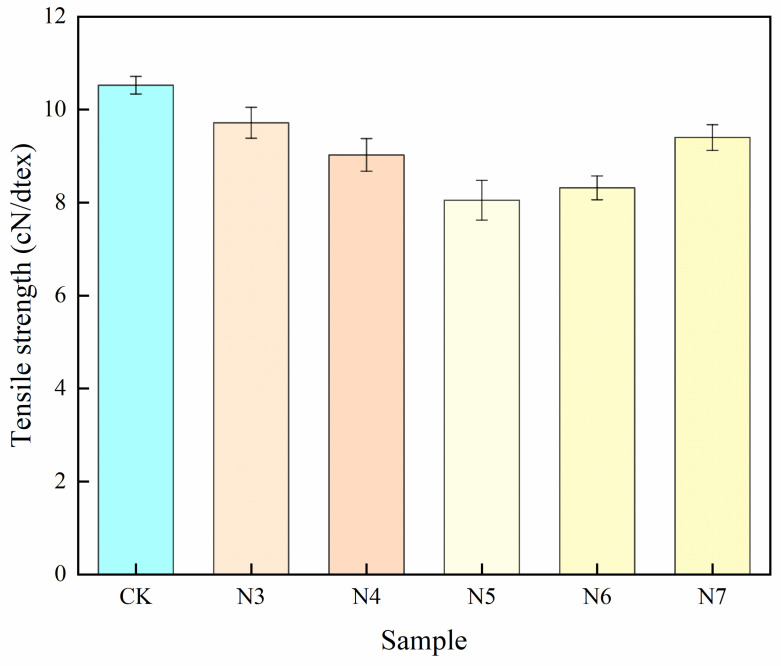
Tensile strength of polyester fabric.

Changes in tensile strength were used to evaluate the mechanical integrity of polyester fibers during microbial degradation, as tensile strength is a sensitive indicator of structural deterioration. Mechanical testing revealed a consistent decrease in tensile strength for all biologically treated samples compared with the control group (CK), indicating progressive damage to the fiber structure (Table 3, Figure 5).

As shown in Figure 5, the tensile strength of the control sample remained at a relatively high level, whereas fibers treated with different bacterial strains exhibited varying degrees of strength loss. Among them, the N5-treated fibers showed the most pronounced reduction, with tensile strength decreasing from 10.53 cN/dtex to 8.05 cN/dtex (a reduction of 23.5%), followed by the N6-treated fibers, which decreased to 8.32 cN/dtex (20.97%). In contrast, the tensile strength reductions for the N3-, N4-, and N7-treated samples were 7.67%, 14.24%, and 10.68%, respectively, demonstrating a clear gradient among different strains. These differences can be attributed to variations in degradation efficiency and interaction mechanisms between bacterial strains and polyester fibers. In particular, strains N5 and N6 induced more pronounced mechanical deterioration, suggesting more effective disruption of the fiber microstructure. During biodegradation, cleavage of ester bonds weakens intermolecular interactions and promotes the formation of microvoids or microcracks, which act as stress concentration sites and accelerate mechanical failure under tensile loading.

**Figure 6:**
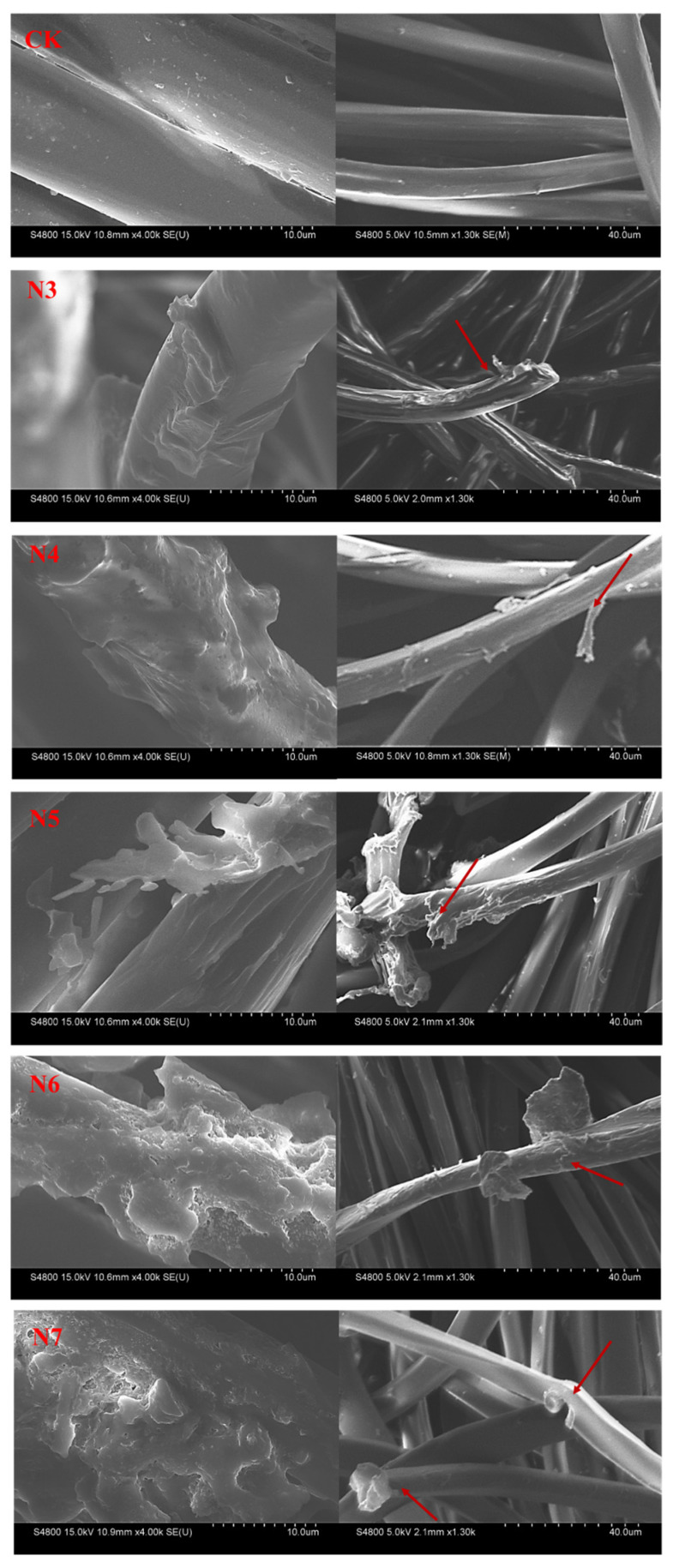
SEM image of polyester fabric, surface covered with microbial film (**left**), microbial film removed (**right**).

Correlation analysis further revealed a strong positive relationship between tensile strength loss and the reduction in number-average molecular weight (M_n_) (R^2^ = 0.91, *p* < 0.01), indicating that partial polymer chain scission contributes to the deterioration of mechanical properties. Notably, although the N3-treated fibers exhibited a relatively low mass loss (2.43%), their tensile strength still decreased markedly. SEM observations (Figure 6) revealed surface grooves on these fibers, suggesting that localized surface erosion and stress concentration, rather than extensive bulk degradation, may play a dominant role in mechanical weakening at this stage.

#### 3.3.3. Surface Morphological Evolution and Wettability Changes of Polyester Fibers

Scanning electron microscopy (SEM) observations revealed that the original polyester fibers had smooth surfaces with no apparent defects, whereas fibers treated with bacterial strains exhibited varying degrees of surface erosion, characterized by grooves and pits on the fiber surface, which is consistent with enzyme-mediated surface attack by extracellular bacterial enzymes (Figure 6). Notably, the fibers degraded by bacterial action were covered by a distinct biofilm, as shown in the image on the left. Biofilm formation by bacteria is typically the initial step in polymer biodegradation [30]. This biofilm is likely composed of extracellular polymeric substances (EPS) and other bioactive compounds secreted by the bacterial strains. In comparison to the control group (CK), the fiber surface covered by the biofilm appeared rougher and more irregular. Such microbial colonization not only significantly reduces the hydrophobicity of the PET surface but also enhances its bioavailability, creating favorable conditions for the subsequent attachment and action of PET-degrading enzymes [31]. In the N5 and N6 treatment groups, the fiber surfaces were fully covered by continuous biofilms. After removing the biofilms, erosion pits with diameters ranging from 1–3 μm were observed, and some areas showed signs of fiber bundle peeling. The N3 treatment group exhibited primarily longitudinal grooves with a maximum width of 1.2 μm. The N4 and N7 treatment groups demonstrated relatively minor surface damage, with only scattered punctate erosion visible. This variation in surface damage may be attributed to the distinct attachment modes and enzyme secretion profiles of the strains—*Bacillus* strains (N5, N6) tend to form biofilms to enhance substrate contact, whereas *Lysodon bacillus* strains (N3, N4) likely achieve localized degradation through motile colonization [32].

The water contact angle (WCA) values of polyester fibers after microbial treatment are summarized in Table 4. The untreated polyester fabric (CK) exhibited typical hydrophobicity, with a contact angle of 125.73 ± 0.70°. Following microbial degradation, the contact angle of polyester fibers decreased to varying extents across the different strains. In the N5 treatment group, the contact angle decreased to 116.46 ± 0.97°, corresponding to a reduction of 7.37%; in the N6 group, it decreased to 119.44 ± 0.62° (a 5.00% reduction); and in the N4 group, it decreased to 121.32 ± 1.30° (a 3.51% reduction). The changes observed in the N3 and N7 groups were relatively minor, with reductions in the range of 1.8–2.3%. Plohl et al. reported a slow reduction in the contact angle of PET fibers, with hydrophobicity decreasing by only 8.6% over six months [33]. The more pronounced decrease in hydrophobicity observed in this study can likely be attributed to two factors: (i) increased surface roughness resulting from surface erosion and (ii) the exposure of polar groups, such as carboxyl groups, generated during degradation, as supported by SEM and FTIR analyses. This observation aligns with the mechanism proposed by Arbade et al., which suggests that biodegradation promotes the surface hydrophilization of materials through the exposure of hydroxyl and carboxyl groups [34].

#### 3.3.4. Changes in Thermal Properties and Crystallization Behavior

**Figure 7:**
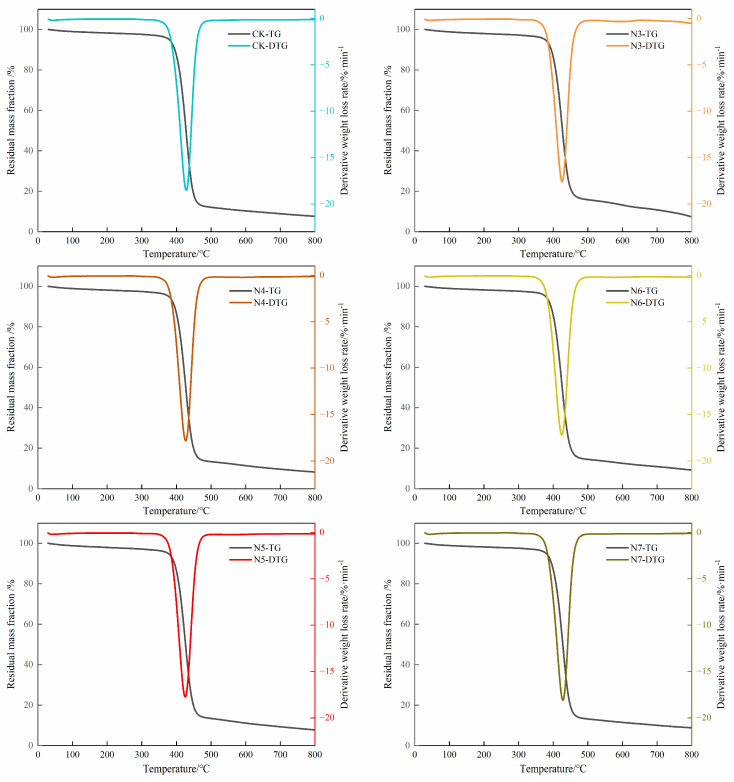
TG-DTG curve analysis of polyester fabric.

To investigate the weight loss behavior of polyester fibers and to infer their thermal stability and decomposition characteristics, thermogravimetric (TG) and differential thermogravimetric (DTG) analyses were performed, and the corresponding curves are shown in Figure 7, with characteristic parameters summarized in Table 5. As observed, the total mass loss of polyester fibers during pyrolysis ranged from approximately 85% to 95%, which is typical for PET materials undergoing thermal decomposition. All samples exhibited a single major weight loss stage with a smooth reverse S-shaped TG curve, reflecting the continuous and consistent nature of the polyester pyrolysis process.

A closer examination of the TG–DTG curves shows that the polyester fibers remained thermally stable up to approximately 350–400 °C, with no pronounced mass loss observed in this temperature range. The main thermal degradation occurred within a relatively narrow temperature window centered around 420–460 °C. The maximum degradation rate temperature (T_p_) of the untreated polyester fiber was 428.46 °C. After bacterial treatment, T_p_ showed slight downward shifts depending on the strain, with values of 424.80 °C for N6 and 425.41 °C for N5 (Table 5). These modest changes suggest minor alterations in thermal behavior, which are consistent with partial molecular chain scission and the presence of low-molecular-weight fragments, rather than pronounced changes in intrinsic thermal stability. The residual mass of the bacterially treated samples also showed limited variation. For example, the residue rate of the N6-treated sample (8.7%) was lower than that of the untreated control (12.3%), indicating a relatively higher degree of volatilization during thermal decomposition, which is consistent with the formation of smaller molecular fragments.

**Figure 8:**
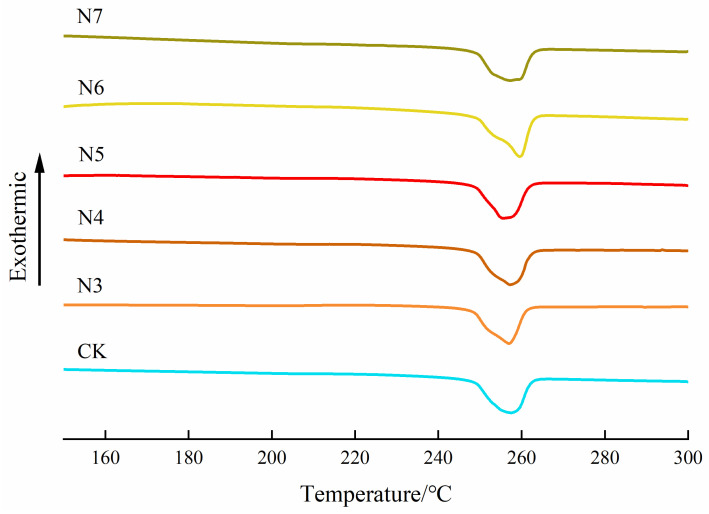
DSC melting curve of polyester fabric.

Differential scanning calorimetry (DSC) analysis revealed a slight increase in the crystallinity (X_c_) of polyester fibers after bacterial treatment (Figure 8, Table 6). Specifically, the crystallinity increased from 41.74% to 43.39% in the N5-treated group, while a smaller increase to 42.00% was observed for the N6-treated sample. Although these changes are relatively modest and occur within a narrow range, they suggest a tendency toward selective structural modification rather than bulk recrystallization.

The observed increase in crystallinity is likely associated with the preferential degradation of amorphous regions, while the crystalline domains remain comparatively stable. Similar behavior has been reported by Spyros and Kimmich, who demonstrated that enzymatic degradation of poly(3-hydroxybutyrate) predominantly occurred in amorphous regions, leading to a passive increase in crystallinity without significant alteration of the crystalline phase [35]. The modest crystallinity changes observed in the present study indicate that a comparable degradation preference may exist for the bacterial strains investigated.

In addition to the main melting peak corresponding to the polyester main chain, a minor melting peak was detected in the temperature range of 245–255 °C for samples treated with strains N3–N7. This feature can be attributed to the melting of oligomeric species generated during microbial degradation, which possess lower molecular weights and consequently lower melting temperatures than the parent polymer. The appearance of these oligomer-related thermal features, despite the minimal changes in overall melting temperature and enthalpy, supports the occurrence of partial chain scission and polymer chain reorganization under low-intensity enzymatic degradation conditions, thereby contributing to a mechanistic understanding of polyester fiber biodegradation by bacterial strains.

### 3.4. Study on the Biodegradation Mechanism of Polyester Fibers

#### 3.4.1. Analysis of Functional Group Changes

**Figure 9:**
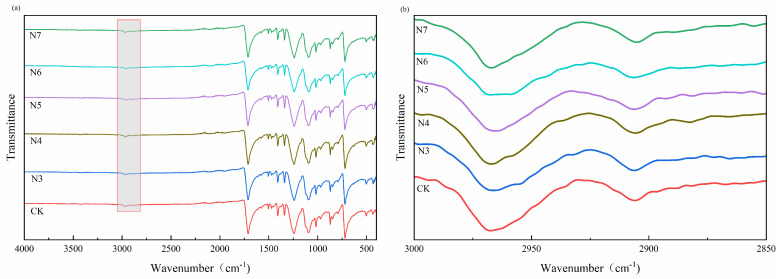
FTIR spectra of polyester fibers after microbial treatment: (**a**) full spectra (4000–500 cm^−1^); (**b**) enlarged view of the methylene (–CH_2_–) stretching region (3000–2850 cm^−1^).

Fourier transform infrared (FTIR) spectroscopy was applied to investigate the functional group variations of polyester fibers before and after microbial treatment, aiming to evaluate the structural response of PET to bacterial degradation at the molecular level. The FTIR spectra of untreated fibers (CK) and fibers treated with strains N3–N7 are presented in Figure 9, including the full spectral range (4000–500 cm^−1^) and an enlarged view of the methylene stretching region (3000–2850 cm^−1^).

As shown in Figure 9a, all samples exhibit the characteristic absorption bands of polyethylene terephthalate (PET), including the ester carbonyl stretching vibration at approximately 1710–1715 cm^−1^, the asymmetric C–O stretching vibration near 1240–1250 cm^−1^, and the C–O–C stretching vibration around 1090 cm^−1^. No obvious peak shifting or new absorption bands were observed after microbial treatment, indicating that the fundamental chemical framework of PET was largely preserved during the degradation process.

To further identify subtle functional group changes induced by microbial action, the methylene (–CH_2_–) stretching region between 3000 and 2850 cm^−1^ was examined in detail (Figure 9b). Compared with the untreated control, all bacteria-treated samples exhibited higher transmittance in this region, corresponding to a decrease in the relative absorbance of –CH_2_– groups associated with the –COO–CH_2_–CH_2_–O– segments of the PET molecular chain. This change suggests that microbial treatment affected the aliphatic components of the polymer structure.

Notably, the extent of methylene-related spectral changes varied among different bacterial strains. The fibers treated with strains N5 and N6 showed more pronounced reductions in –CH_2_– absorption compared with N3 and N4, indicating stronger interactions between these strains and the polyester substrate. In particular, the N6-treated samples exhibited the most evident change in the methylene stretching region, suggesting a higher degree of functional group modification.

The hydroxyl (–OH) stretching band in the range of 3200–3600 cm^−1^ was not clearly detected in the spectra, indicating that the amount of hydroxyl-containing species generated during microbial treatment was limited and below the detection sensitivity of FTIR under the experimental conditions.

Overall, FTIR analysis demonstrates that microbial treatment induced detectable variations in methylene-related functional groups while maintaining the overall chemical structure of PET. These functional group changes provide molecular-level evidence that the polymer chains were structurally perturbed by microbial activity, thereby laying a structural basis for the formation of degradation products identified in subsequent GCMS analysis.

#### 3.4.2. Identification of Degradation Products and Pathway Analysis

**Figure 10:**
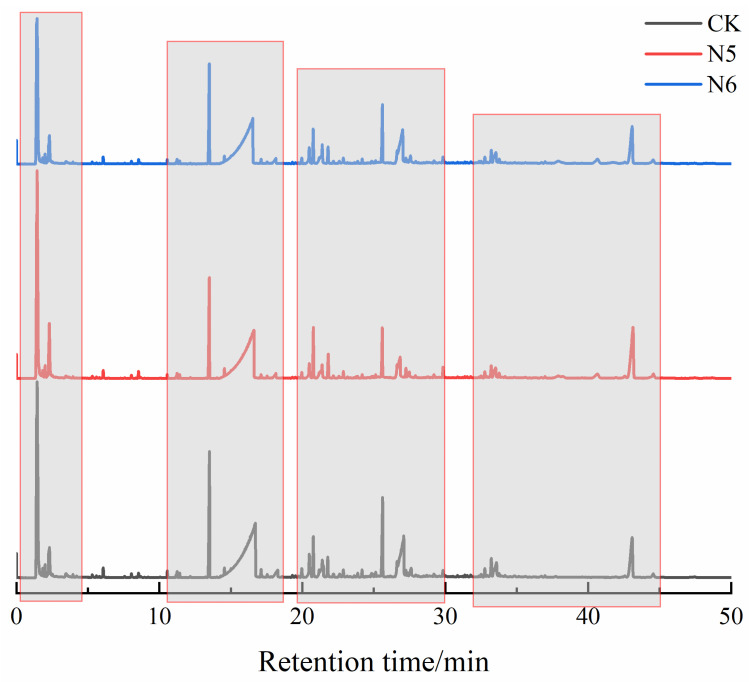
GCMS curve analysis of polyester fabric.

**Figure 11:**
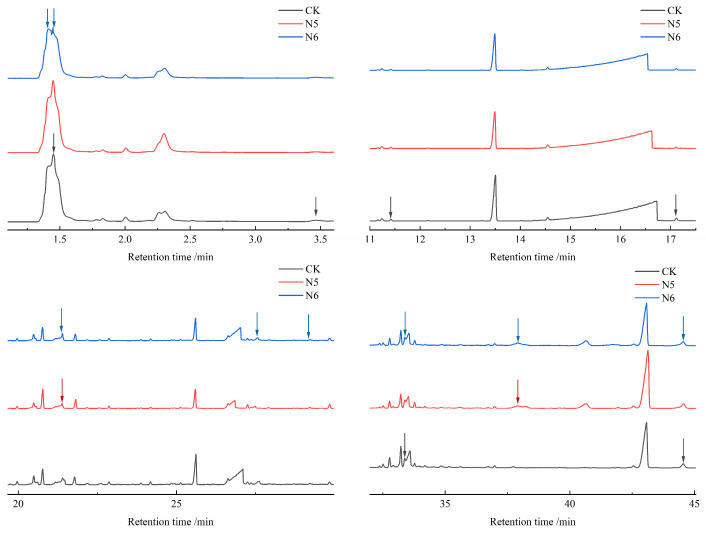
GCMS local magnification curve analysis of polyester fiber fabric.

To further investigate the effects of strains N5 and N6 on the generation of low-molecular-weight degradation products of polyester fibers, GC–MS analysis was conducted on the solvent-extractable degradation products of the fibers after microbial treatment. Figure 10 and Figure 11 present the chromatograms of polyester fibers, highlighting differences in peak intensities corresponding to various compounds at distinct retention times. The untreated polyester fibers (CK) mainly exhibited characteristic peaks at retention times of 1.451 min, 16.720 min, and 27.095 min. In contrast, fibers treated with strain N5 showed peaks at 1.449 min, 16.621 min, and 26.845 min, while those treated with N6 appeared at 1.413 min, 16.535 min, and 27.025 min. The shift of several characteristic peaks toward shorter retention times suggests that microbial treatment facilitated polymer chain scission and the formation of smaller, more volatile degradation products, indicating reduced molecular integrity of the polyester fibers.

During microbial degradation of PET, enzymatic hydrolysis of ester bonds induces chain scission and produces oligomeric intermediates. According to previous studies, ester bond cleavage may proceed through β-scission-related pathways, resulting in the formation of carboxyl-terminated and vinyl ester-terminated fragments [36]. Typical degradation products such as bis(2-hydroxyethyl) terephthalate (BHET) and terephthalic acid (TPA) were detected (Supplementary Tables S1–S3), confirming the occurrence of ester bond hydrolysis. Notably, benzoic acid, with a relative content of 35.42–36.71%, a characteristic intermediate of PET degradation, was detected in all microbial treatment groups. Additionally, mono(2-hydroxyethyl) terephthalate (MHET) and TPA were identified in the N5 and N6 groups, further confirming that enzymatic hydrolysis of ester bonds generated common PET degradation products.

Strikingly, benzoic acid 2-chloroethyl ester (0.64%) was detected in the N5 treatment group, and isophthalic acid (0.51%) was detected in the N6 group. These compounds are indicative of the degradation or transformation of additives such as plasticizers and dyes present in the fibers, suggesting that strains N5 and N6 are capable of simultaneously degrading both the PET backbone and additive components.

Compared with the control group, the relative content of BHET decreased from 0.43% to 0.36–0.40% in the N5 and N6 groups, while TPA decreased from 0.82% to 0–0.30%. Under the action of strains N5 and N6, the ester bonds in BHET were further cleaved. Notably, TPA was not detected in the N6-treated group, suggesting that PETase catalyzed the conversion of BHET into MHET, which was subsequently hydrolyzed by MHETase into TPA and ethylene glycol (EG). The absence of detectable EG may be attributed to its further metabolism or secondary reactions, such as dehydration, decarboxylation, or condensation processes leading to small molecular products.

In summary, strains N5 and N6 preferentially degrade ester bonds and adjacent aliphatic segments of polyester fibers through enzymatic activity, promoting depolymerization and subsequent metabolism of degradation intermediates. The diversity of detected degradation products reflects the complexity of microbial degradation pathways, including main-chain scission, aromatic structure modification, and additive transformation, indicating that N5 and N6 possess strong metabolic capacities enabling more complete degradation of polyester fibers.

#### 3.4.3. Analysis of Degradation Mechanisms

**Figure 12:**
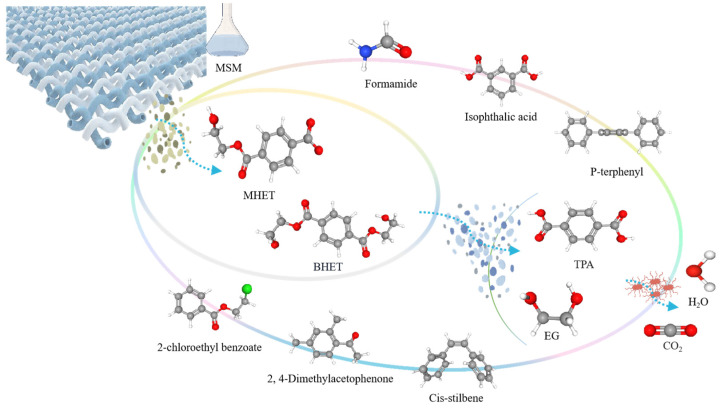
Proposed degradation pathway of polyester fibers mediated by bacterial activity based on FTIR and GC-MS analyses. In the molecular structures, carbon atoms are shown in gray, oxygen atoms in red, hydrogen atoms in white, nitrogen atoms in blue, and chlorine atoms in green.

Based on the combined results of FTIR functional group analysis and GC–MS identification of microbial degradation products, a proposed degradation mechanism for the microbial degradation of polyester fibers is summarized and schematically illustrated in Figure 12.

The degradation process is initiated at the fiber surface, where microbial colonization and enzymatic secretion induce localized structural perturbations. As evidenced by FTIR analysis, microbial treatment leads to detectable changes in methylene (–CH_2_–) related functional groups while preserving the overall PET chemical framework. These observations indicate that degradation begins with surface erosion and preferential attack on structurally accessible aliphatic segments adjacent to ester linkages, rather than uniform bulk chain scission.

Following this initial structural perturbation, ester bonds within the PET molecular chain become susceptible to enzymatic hydrolysis. PET hydrolases, such as PETase, catalyze the cleavage of ester linkages, resulting in depolymerization into soluble intermediates including bis(2-hydroxyethyl) terephthalate (BHET), mono(2-hydroxyethyl) terephthalate (MHET), terephthalic acid (TPA), and ethylene glycol (EG). This stage represents the primary depolymerization step, transforming the insoluble polymer into metabolizable low-molecular-weight compounds, as supported by GC–MS analysis of extractable degradation products.

Importantly, the accumulation and subsequent transformation of these intermediates play a critical role in controlling the overall degradation efficiency. Previous studies have demonstrated that intermediates such as BHET and MHET can inhibit the activity of certain PET hydrolases due to their structural similarity to PET ester bonds, thereby limiting depolymerization rates [37].

However, specific enzymes, including HiC and Cut190, have been reported to overcome this inhibitory effect, enabling further hydrolysis of MHET into TPA and EG. The reduced abundance or absence of key intermediates, particularly in the N6 treatment group, suggests that this strain possesses enhanced metabolic capabilities for intermediate conversion, facilitating more continuous depolymerization and downstream metabolism.

In addition to PET backbone depolymerization, the presence of additives and auxiliary components in polyester fabrics introduces parallel degradation pathways. Enzymatic and oxidative processes facilitate the transformation of aromatic additives and plasticizers, generating secondary products such as substituted benzoic acid derivatives and aromatic esters. These reactions reflect the broad substrate specificity of microbial enzymes and further contribute to the chemical complexity of the degradation system.

Ultimately, the combined action of surface erosion, enzymatic depolymerization, and subsequent metabolic conversion of intermediates drives the progressive breakdown of polyester fibers. Aromatic and aliphatic fragments undergo further oxidation, hydrolysis, and rearrangement reactions, leading toward mineralization into CO_2_ and H_2_O. The more pronounced functional group alterations observed by FTIR and the diversified degradation products identified by GC–MS for the N6 strain indicate that this strain achieves a higher degree of degradation through coordinated depolymerization and efficient intermediate utilization.

Overall, the proposed mechanism highlights that microbial degradation of polyester fibers proceeds through a combined surface erosion–bulk depolymerization pathway, governed not only by ester bond cleavage but also by the efficiency of intermediate removal and metabolic integration. This mechanistic framework explains the strain-dependent degradation behaviors observed in this study and provides insight into microbial strategies for the effective biodegradation of polyester materials.

## 4. Conclusions

This study systematically evaluated the biodegradation potential of environmental *Bacillus* strains toward high-crystallinity polyester fibers under mild, mesophilic conditions without external carbon sources. Several strains, particularly N5 and N6, exhibited measurable degradation activity, demonstrating the capacity of *Bacillus* species to partially depolymerize industrial-grade polyester fibers under environmentally relevant conditions.

Integrated multi-scale characterization revealed that polyester fiber biodegradation is a progressive and coordinated process involving enzyme-mediated surface erosion accompanied by partial bulk depolymerization of the polymer backbone, followed by the transformation of low-molecular-weight intermediates. Surface morphological alterations, molecular weight reduction, tensile strength loss, and changes in thermal and surface properties consistently indicate preferential degradation of amorphous regions while largely preserving the crystalline domains. GC–MS analysis provided molecular-level evidence for ester bond cleavage and the formation of characteristic degradation intermediates, highlighting the importance of intermediate utilization in sustaining degradation efficiency.

Notably, strain-dependent differences were observed, with N5 and N6 exhibiting stronger surface modification and intermediate turnover capabilities, whereas N4 showed relatively higher activity toward pure PET substrates. These results suggest functional complementarity among *Bacillus* strains and imply that rationally designed microbial consortia may enhance polyester fiber biodegradation.

Overall, this study provides microbial resources and mechanistic insight into the biodegradation of high-crystallinity polyester fibers under mild conditions. The findings contribute to a clearer understanding of strain-specific degradation behaviors and support the development of low-energy, biologically driven strategies for polyester fiber waste management.

## Figures and Tables

**Table 1:** Results of strain identification.

Sample	Name
C1	*Bacillus aerius*
C2	*Bacillus haynesii*
C3	*Bacillus paralicheniformis*
C4	*Bacillus licheniformis*
C5	*Bacillus paralicheniformis*
C6	*Bacillus licheniformis*
N1	*Lysinibacillus*
N2	*Bacillus licheniformis*
N3	*Lysinibacillus macrolides*
N4	*Lysinibacillus xylanilyticus*
N5	*Bacillus altitudinis*
N6	*Bacillus subtilis*
N7	*Bacillus licheniformis*

**Table 2:** Comparison of the 30-day degradation efficiency of polyester fibers by bacterial strains.

Strain	Loss Rate (%)	Daily Degradation Rate (d^−1^)	Half-Life (d)
N3	2.43 ± 0.18	0.00082	845.07
N4	3.21 ± 0.21	0.00110	630.13
N5	5.15 ± 0.32	0.00176	393.83
N6	5.00 ± 0.29	0.00172	403.01
N7	2.14 ± 0.15	0.00073	950.96
CK	0.14 ± 0.03	-	-

**Table 3:** Changes in mechanical properties after degradation of polyester fibers.

	Tensile Strength (cN/dtex)	Decrease (%)
CK	10.53 ± 0.19	-
N3	9.71 ± 0.33	7.67
N4	9.02 ± 0.35	14.24
N5	8.05 ± 0.42	23.50
N6	8.32 ± 0.26	20.97
N7	9.40 ± 0.28	10.68

**Table 4:** Water contact angle (WCA) of polyester fibers after microbial treatment.

Sample	Water Contact Angle (°)
CK	125.73 ± 0.70
N3	123.57 ± 0.56
N4	121.32 ± 1.30
N5	116.46 ± 0.97
N6	119.44 ± 0.62
N7	124.68 ± 0.83

**Table 5:** Thermal decomposition characteristics of polyester fabric.

Sample	DTG_peak_/(%·min^−1^)	T_p_/°C	T_i_/°C	T_c_/°C
PET	−18.51	428.46	399.49	459.73
N3 + PET	−17.60	425.69	396.55	458.01
N4 + PET	−17.81	426.84	397.55	459.58
N5 + PET	−17.74	425.41	392.82	458.70
N6 + PET	−17.22	424.80	393.11	456.80
N7 + PET	−18.10	427.96	398.04	460.13

Note: T_p_ is the maximum weight loss rate temperature, T_i_ is the weight loss start temperature, and T_c_ is the epitaxial termination temperature.

**Table 6:** DSC melting parameters of polyester fabric.

Sample	T_a_/°C	T_m_/°C	T_b_/°C	ΔH_m_/J·g^−1^	X_c_/%
PET	247.99	256.90	261.87	54.35	41.74
N3 + PET	247.80	256.62	260.97	51.85 ↓	39.82
N4 + PET	248.38	256.55	261.87	54.22 ↓	41.64
N5 + PET	248.79	254.96	261.55	56.50 ↑	43.39
N6 + PET	251.38	258.66	262.13	54.68 ↑	42.00
N7 + PET	248.32	256.53	262.23	52.92 ↓	40.65

Note: T_a_ is the initial temperature, T_m_ is the peak temperature, T_b_ is the final temperature, ΔH_m_ is the melting enthalpy, and X_c_ is the crystallinity. Arrows indicate an increase (↑) or decrease (↓) in ΔH_m_ compared with the PET sample.

## Data Availability

The original contributions presented in this study are included in the article/Supplementary Material. Further inquiries can be directed to the corresponding authors.

## Supplementary Materials

The following supporting information can be downloaded at: https://www.mdpi.com/article/10.3390/microorganisms14010207/s1, Figure S1: Optical density (OD600) profiles of Bacillus strains cultured with polyester fibers; Table S1: Major pyrolysis products detected from untreated polyester fabric at 550 °C; Table S2: Major pyrolysis products detected from polyester fabric degraded by Bacillus strain N5 at 550 °C; Table S3: Major pyrolysis products detected from polyester fabric degraded by Bacillus strain N6 at 550 °C.
