# Supplementary material for: Screening, Identification, and Degradation Mechanism of Polyester Fiber-Degrading Bacteria
Source: Microorganisms. 2026 Jan 16;14(1):207. doi: 10.3390/microorganisms14010207 (PMC12843709; doi:10.3390/microorganisms14010207)
Supplement: Supplementary file 1 [file microorganisms-14-00207-s001.zip › microorganisms-4087470-supplementary.pdf]

Supplementary Material

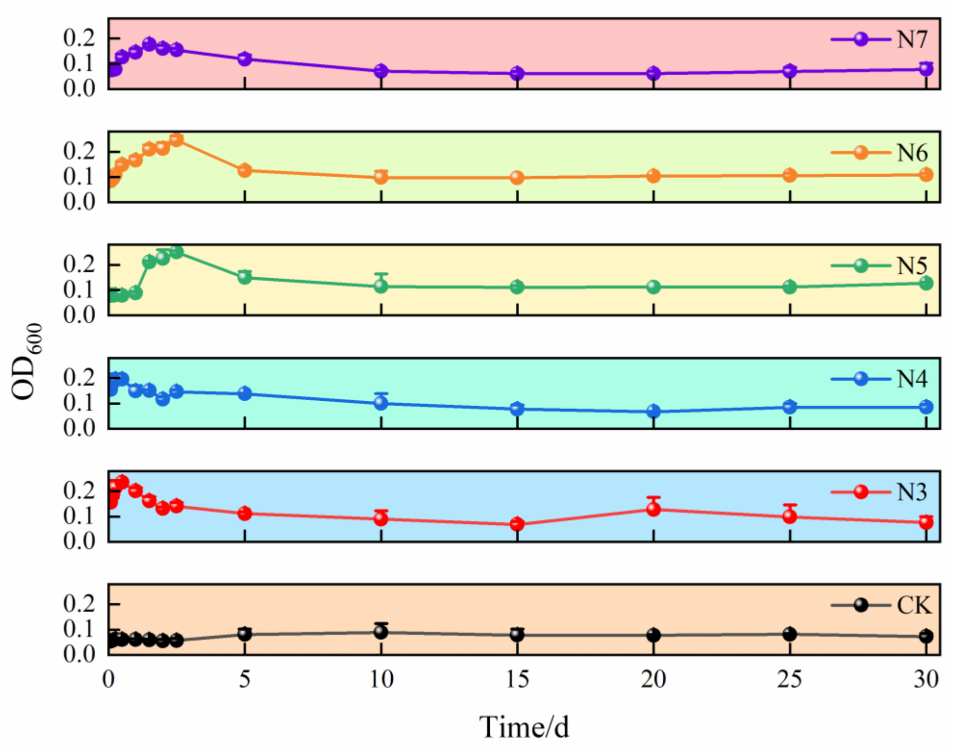

Figure S1. Optical density (OD600) profiles of Bacillus strains cultured with polyester fibers.

Detailed results of the pyrolysis–gas chromatography/mass spectrometry (Py–GC/MS) analysis are provided below.

These tables summarize the major pyrolysis products identified from untreated and biodegraded polyester fabrics at 550 °C.

Each table lists the peak number, molecular formula, retention time (min), relative peak area (%), and compound name.

These data provide detailed chemical evidence of the thermal decomposition behavior of polyester fibers before and after microbial degradation.

Table S1. Major pyrolysis products detected from untreated polyester fabric at 550 °C.

Peak No.	MolecularFormula	Retention time(min)	Relative peak area (%)	Compound Name
1	CH_6Si	1.451	18.13	Methylsilane
2	C_4H_6O	1.777	0.39	2,3-Dihydrofuran
3	C_4H_8O	1.832	0.28	Butyraldehyde
4	C_4H_8O	2.006	0.39	Tetrahydrofuran
5	C_6H_6	2.308	2.2	Benzene
6	C_7H_8	3.467	0.22	Toluene
7	C_8H_8	6.069	0.41	Styrene

Peak No.	MolecularFormula	Retention time(min)	Relative peak area (%)	Compound Name
8	C_7H_6O	8.044	0.16	Benzaldehyde
9	C_6H_6O	8.542	0.19	Phenol
10	C_8H_8O	10.559	0.24	Phenylacetaldehyde
11	C_8H_8O	11.244	0.24	Acetophenone
12	$C_{10}H_{10}O_3$	11.422	0.15	-
13	$C_9H_8O_2$	13.505	6.3	Vinyl benzoate
14	$C_9H_{10}O_2$	14.551	0.26	Ethyl benzoate
15	$C_7H_6O_2$	16.72	35.44	Benzoic acid
16	C8H7C10	17.115	0.28	o-Toluoyl chloride
17	$C_8H_8O_2$	18.279	1.05	p-Toluic acid
18	$C_{12}H_{14}N_4O$	19.965	0.37	-
19	$C_{11}H_{10}O_2$	20.49	0.97	Vinyl cinnamate
20	$C_9H_{10}O_2$	20.617	0.36	p-Ethylbenzoic acid
21	$C_{12}H_{10}$	20.77	1.81	Biphenyl
22	$C_8H_5BrO_2$	21.181	0.14	3-Bromophthalide
23	$C_8H_7NO_2$	21.402	1.02	2-Nitrosostilbene
24	$C_9H_{10}O_3$	21.782	1	2-Hydroxyethyl benzoate
25	$C_{13}H_{12}$	22.172	0.22	Diphenylmethane
26	$C_{10}H_{10}O_2$	22.622	0.24	2,3-Dihydro-1H-indene-5-carboxylic acid
27	$C_{18}H_{18}O_5$	22.871	0.31	Di(ethylene glycol) dibenzoate
28	$C_{13}H_{12}$	23.878	0.17	3-Methylbiphenyl
29	$C_{10}H{=}_{10}O_2$	24.182	0.3	1,4-Diacetylbenzene
30	$C_{17}H_{15}NO_2$	24.846	0.19	4-Cyanophenyl 4-propylbenzoate
31	$C_8H_{12}O_3$	24.95	0.13	Tetrahydrofurfuryl acrylate
32	$C_{12}H_{24}O_3$	25.134	0.21	1,6,11-Trioxacyclopentadecane
33	$C_{11}H_{12}N_2O_2$	25.618	4.17	Phenylethylhydantoinoin
34	$C_{11}H_{12}O_3$	26.633	1.02	Ethyl 4-acetylbenzoate
35	$C_{10}H_{10}O_3$	27.095	9.22	o-Propionylbenzoic acid
36	$C_{13}H_{10}O$	27.251	0.33	Benzophenone
37	$C_{14}H_{12}$	27.363	0.19	4-Vinylbiphenyl
38	$C_8H_6O_4$	27.618	0.82	Terephthalic acid
39	$C_{13}H_8O$	29.842	0.34	Benzophenone
40	$C_{13}H_{12}N_2O$	32.514	0.15	4-Biphenylcarbohydrazide
41	C12H14O6	32.773	0.43	Bis(2-hydroxyethyl) terephthalate
42	$C_{13}H_{12}N_2O$	33.226	1.01	4-Biphenylcarbohydrazide
43	$C_{19}H_{18}F_2O_4$	33.377	0.56	-
44	$C_{13}H_{10}O_2$	33.588	1.58	4-Biphenylbenzoic acid
45	$C_{14}H_{10}O_3$	33.778	0.17	Benzoic anhydride
46	$C_{16}H_{14}O_4$	43.078	5.84	Diethyl benzoate
47	$C_{18}H_{14}$	44.557	0.39	m-Terphenyl

Table S2. Major pyrolysis products detected from polyester fabric degraded by Bacillus strain N5 at 550 °C.

Peak No.	MolecularFormula	Retention time(min)	Relative peak area (%)	Compound name
1	CH_6Si	1.449	21.13	Methylsilane
2	C_4H_6O	1.773	0.41	2,3-Dihydrofuran
3	C_4H_8O	1.83	0.29	Butyraldehyde
4	C_4H_8O	2.008	0.47	Tetrahydrofuran
5	C_6H_6	2.3	3.89	Benzene
6	C_8H_8	6.068	0.4	Styrene
7	C_7H_6O	8.044	0.17	Benzaldehyde
8	C_6H_6O	8.542	0.35	Phenol
9	C_8H_8O	10.557	0.18	Phenylacetaldehyde
10	C_8H_8O	11.243	0.21	Acetophenone
11	$C_9H_8O_2$	13.493	5.52	Vinyl benzoate
12	$C_9H_{10}O_2$	14.551	0.35	Ethyl benzoate
13	$C_7H_6O_2$	16.621	35.42	Benzoic acid
14	$C_8H_8O_2$	18.151	0.56	p-Toluic acid
15	-	19.964	0.29	-
16	$C_{11}H_{10}O_2$	20.489	0.76	Vinyl cinnamate
17	$C_9H_{10}O_2$	20.567	0.16	p-Ethylbenzoic acid
18	$C_{12}H_{10}$	20.774	2.64	Biphenyl
19	$C_8H_5BrO_2$	21.173	0.98	3-Bromophthalide
20	$C_9H_9ClO_2$	21.385	0.64	2-Chloroethyl benzoate
21	$C_9H_{10}O_3$	21.817	1.33	2-Hydroxyethyl benzoate
22	$C_{18}H_{18}O_5$	22.873	0.28	Di(ethylene glycol) dibenzoate
23	$C_{10}H_{10}O_2$	24.187	0.16	1,4-Diacetylbenzene
24	$C_{11}H_{12}N_2O_2$	25.598	2.74	Phenylethylhydantoin
25	$C_{11}H_{12}O_3$	26.634	1.09	Ethyl 4-acetylbenzoate
26	$C_{10}H_{10}O_3$	26.845	2.72	o-Propionylbenzoic acid
27	$C_{13}H_{10}O$	27.251	0.47	Benzophenone
28	$C_{14}H_{12}$	27.353	0.16	4-Vinylbiphenyl
29	$C_8H_6O_4$	27.492	0.3	Terephthalic acid
30	$C_{13}H_8O$	29.844	0.64	Benzophenone
31	$C_{12}H_{14}O_6$	32.768	0.36	Bis(2-hydroxyethyl) terephthalate
32	$C_{13}H_{12}N_2O$	33.217	0.6	4-Biphenylcarbohydrazide
33	-	33.373	0.65	-
34	$C_{13}H_{10}O_2$	33.525	0.78	4-Biphenylbenzoic acid
35	$C_{14}H_{10}O_3$	33.774	0.28	Benzoic anhydride
36	$C_{10}H_{12}O$	37.896	0.93	3,4-Dimethylacetophenone
37	$C_{11}H_{10}O_2$	40.647	1.1	Vinyl cinnamate
38	$C_{16}H_{14}O_4$	43.136	9.82	Diethyl benzoate
39	$C_{18}H_{14}$	44.568	0.77	p-Terphenyl

Table S3. Major pyrolysis products detected from polyester fabric degraded by Bacillus strain N6 at 550 °C.

Peak No.	MolecularFormula	Retention time(min)	Relative peak area (%)	Compound name
1	CH_3NO	1.413	9.4	Formamide
2	-	1.447	9.65	-
3	C_4H_6O	1.77	0.1	2,3-Dihydrofuran
4	C_4H_8O	1.826	0.17	Butyraldehyde
5	C_4H_8O	2.002	0.34	Tetrahydrofuran
6	C_6H_6	2.303	2.45	Benzene
7	C_8H_8	2.603	0.39	Styrene
8	C_7H_6O	8.04	0.13	Benzaldehyde
9	C_6H_6O	8.544	0.22	Phenol
10	C_8H_8O	10.557	0.21	Phenylacetaldehyde
11	C_8H_8O	11.239	0.22	Acetophenone
12	-	11.42	0.12	-
13	$C_9H_8O_2$	13.492	6.07	Vinyl benzoate
14	$C_9H_{10}O_2$	14.547	0.27	Ethyl benzoate
15	$C_7H_6O_2$	16.535	36.71	Benzoic acid
16	C8H7ClO	17.113	0.21	o-Methylbenzoyl chloride
17	$C_8H_8O_2$	18.149	0.75	p-Toluic acid
18	-	19.96	0.31	-
19	$C_{11}H_{10}O_2$	20.484	0.91	Vinyl cinnamate
20	$C_9H_{10}O_2$	20.56	0.14	p-Ethylbenzoic acid
21	$C_{12}H_{10}$	20.764	2	Biphenyl
22	$C_8H_5BrO_2$	21.183	0.1	3-Bromophthalide
23	$C_9H_9ClO_2$	21.396	0.66	2-Chloroethyl benzoate
24	$C_9H_{10}O_3$	21.801	0.98	2-Hydroxyethyl benzoate
27	$C_{11}H_{12}N_2O_2$	25.604	3.68	Phenylethylhydantoinn
28	$C_{11}H_{12}O_3$	26.634	0.73	Ethyl 4-acetylbenzoate
29	$C_{10}H_{10}O_3$	27.025	7.99	o-Propionylbenzoic acid
30	$C_{13}H_{10}O$	27.249	0.23	Benzophenone
31	$C_{14}H_{12}$	27.366	0.12	4-Vinylbiphenyl
32	$C_8H_6O_4$	27.572	0.51	Isophthalic acid
33	C14H12	29.218	0.11	cis-Stilbene
34	C13H8O	29.844	0.41	Benzophenone
35	C13H12N2O	32.515	0.14	4-Biphenylcarbohydrazide
36	$C_{12}H_{14}O_6$	32.774	0.4	Bis(2-hydroxyethyl) terephthalate
37	$C_{13}H_{12}N_2O$	33.222	0.78	4-Biphenylcarbohydrazide
38	-	33.384	0.5	-
39	$C_{13}H_{10}O_2$	33.541	1.38	4-carboxybiphenyl
40	$C_{14}H_{10}O_3$	33.779	0.31	Benzoic anhydride
41	$C_{10}H_{12}O$	37.907	0.95	2,4-Dimethylacetophenone
42	$C_{11}H_{10}O_2$	40.643	1.3	Vinyl cinnamate
43	$C_{16}H_{14}O_4$	43.08	7	Diethyl benzoate
44	$C_{18}H_{14}$	44.552	0.49	p-Terphenyl

Note: Compound identification was performed by comparison with the NIST 17 mass spectral database.

The results confirm that microbial pretreatment (N5 and N6) alters the pyrolysis profile of polyester fibers, leading to earlier thermal decomposition and formation of additional aromatic derivatives.
